# Leukocyte migration in experimental inflammatory bowel disease 

**DOI:** 10.1080/09629359791776

**Published:** 1997-04

**Authors:** E. P. Van Rees, M. J. H. J. Palmen, F. R. W. Van De Goot, B. A. Macher, L. A. Dieleman

**Affiliations:** 1 Department of Cell Biology and Immunology Faculty of Medicine Vrije Universiteit Van der Boechorststraat 7 Amsterdam 1081 BT The Netherlands; 2 Department of Chemistry and Biochemistry San Francisco State University San Francisco USA; 3 Division of Digestive Disease and Nutrition University of North Carolina School of Medicine Chapel Hill NC USA

## Abstract

Emigration of leukocytes from the circulation into tissue by transendothelial migration, is mediated subsequently by adhesion molecules such as selectins, chemokines and integrins. This multistep paradigm, with multiple molecular choices at each step, provides a diversity in signals. The influx of neutrophils, monocytes and lymphocytes into inflamed tissue is important in the pathogenesis of chronic inflammatory bowel disease. The importance of each of these groups of adhesion molecules in chronic inflammatory bowel disease, either in human disease or in animal models, will be discussed below. Furthermore, the possibilities of blocking these different steps in the process of leukocyte extravasation in an attempt to prevent further tissue damage, will be taken into account.


Mediators of Inflammation, 6, 85–93 (1997)

### Leukocyte migration in experimental inflammatory bowel disease

E. P. van Rees,[1,CA] M. J. H. J. Palmen,[1] F. R. W. van de Goot,[1] B. A. Macher[2] and L. A. Dieleman[3]

[1]Department of Cell Biology and Immunology, Faculty of Medicine, Vrije Universiteit, Van der Boechorststraat 7, 1081 BT Amsterdam, The Netherlands; [2]Department of Chemistry and Biochemistry, San Francisco State University, San Francisco, USA; [3]Division of Digestive Disease and Nutrition, University of North Carolina School of Medicine, Chapel Hill, NC, USA

[CA]Corresponding Author
Tel: (+31) 20 4448080
Fax: (+31) 20 4448081
Email: e.van_rees.cell@med.vu.nl

**EMIGRATION of leukocytes from the circulation into tissue by transendothelial migration, is mediated subsequently by adhesion molecules such as selectins, chemokines and integrins. This multi-step paradigm, with multiple molecular choices at each step, provides a diversity in signals. The influx of neutrophils, monocytes and lymphocytes into inflamed tissue is important in the pathogenesis of chronic inflammatory bowel disease. The importance of each of these groups of adhesion molecules in chronic inflammatory bowel disease, either in human disease or in animal models, will be discussed below. Furthermore, the possibilities of blocking these different steps in the process of leukocyte extravasation in an attempt to prevent further tissue damage, will be taken into account.**

**Key words:** Animal model, Chemokines, Inflammatory bowel disease, Integrins, Selectins

### Introduction

Leukocyte migration into inflamed tissue is mediated by different groups of adhesion molecules, present on both the endothelial cells and on the leukocytes. This allows tissue-specific leukocyte–endothelial interactions, necessary for efficient surveillance of tissues for infectious pathogens. However, in case of a chronic inflammatory reaction, the accumulation of leukocytes is not the result of an adequate reaction to a pathogenic micro-organism. It is the result of an unbalanced reaction that leads to tissue damage and disease, such as chronic inflammatory bowel disease (IBD).

IBD consists of two major illnesses, ulcerative colitis (UC) and Crohn's disease (CD). These are chronic inflammatory disorders of the intestine of unknown origin. Both diseases primarily affect young adults, but they may present at all ages. In both diseases the inflammatory lesions are localized in the intestine. Both CD and UC exhibit features of chronic inflammation, with prolonged clinical courses and inflammatory infiltrates consisting of lymphocytes and macrophages. However, both diseases also exhibit an acute component, marked by a constant flow of neutrophils out of the circulation into the inflamed mucosa, through the epithelium and into the intestinal lumen.

Despite the fact that the aetiology of IBD remains unclear, advances in research have identified immunological, genetic and environmental factors that could play a role in the aetiology and pathogenesis of these entities. To obtain more insight into the pathogenesis of IBD, animal models are required. An ideal model would be comparable with human disease in pathogenesis, histopathology, and it should respond in a similar way to well-tested therapeutics as human disease. In this sense, neither of the currently available models are ideal for human IBD, yet they can provide useful information about the contribution of the different components of the immune system during intestinal inflammation. They are particularly useful in studying early inflammatory events, and in the identification of immunologic processes and genes that determine susceptibility. Currently the most widely used model is the one originally described by Morris *et al.*[1] In this animal model, colitis is being induced in mice, rat or rabbits by intracolonic administration of the contact sensitizing hapten trinitrobenzene

© 1997 Rapid Science Publishers sulphonic acid (TNBS), dissolved in ethanol. The ethanol is given to break the mucosal barrier and it allows the hapten to enter the intestinal wall. Administration of TNBS in ethanol results in acute inflammation which usually is transmural. Spontaneous healing occurs in approximately 8 weeks. Spontaneous relapses, a characteristic feature in human IBD, are not observed in this model. Histopathological features are discrete patches with acute inflammation and necrosis, followed by a chronic inflammation with a mononuclear cell infiltrate. TNBS-induced colitis seems to be based on a classical delayed-type hypersensitivity response to this hapten. A histopathologic feature of IBD and TNBS/ethanol induced experimental IBD, is a dense infiltration of the tissue by neutrophils and macrophages and, in chronic disease, of lymphocytes.

In IBD, immunoreactivity in the gut is not an adequate, protective response to a known pathogen, but it causes an ongoing inflammatory process that causes severe tissue damage and morbidity. Therefore, blocking the influx of leukocytes into the intestinal wall may prevent further tissue destruction. Emigration of leukocytes from the circulation into tissue, by trans-endothelial migration, is mediated subsequently by adhesion molecules such as selectins, chemokines and integrins.[2] This multistep paradigm, with multiple molecular choices at each step, provides a diversity in signals.[2] The influx of neutrophils and monocytes into inflamed tissue is important in the pathogenesis of IBD. The importance of each of these steps in IBD, either human disease or animal model, and the possibilities for blocking each particular step, will be discussed below.

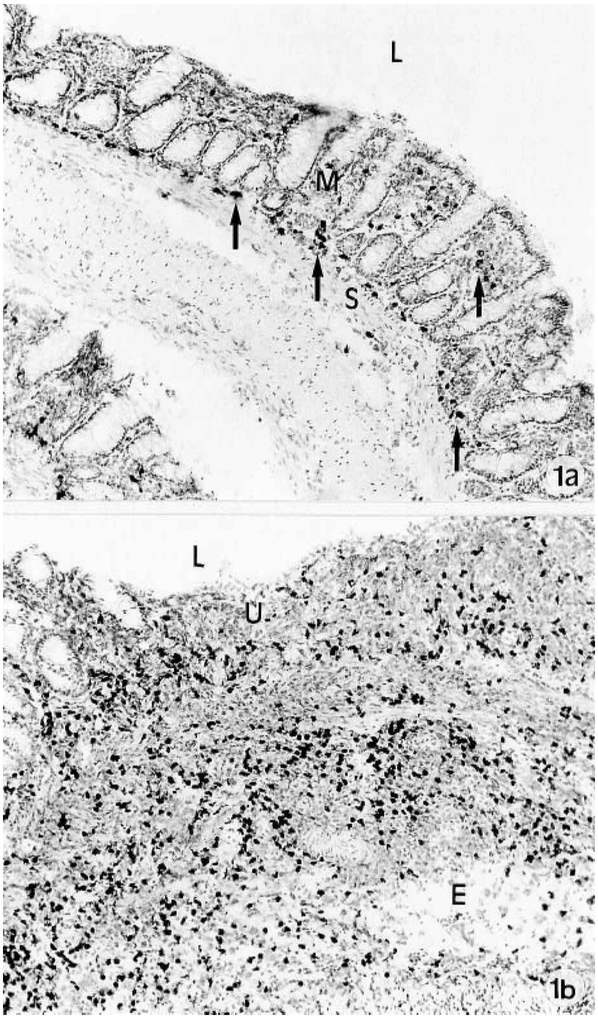

FIG. 1. After induction of colitis by a single enema with TNBS dissolved in ethanol, leukocytes were studied using immunohistochemistry on cryostat sections. (A) Section of colonic tissue obtained from a healthy control rat. Darkly stained neutrophils (arrows) are found in low numbers. M = mucosa; S = submucosa, L = gut lumen. (B) Tissue section obtained at day 7 after induction of colitis. The intestinal wall is transmurally inflamed, a large ulcer is present. Many neutrophils are being found. Oedema is seen as well. U = ulcus; L = gut lumen; E = oedema.

### Influx of Leukocytes in Acute, Chronic and Relapsing Experimental Colitis

In acute experimental colitis in rats, induced by an enema of TNBS in ethanol, neutrophils and macrophages play an important role (Fig. 1) whereas in the chronic stage of the disease T cells play a pivotal role (Fig. 2A).[3] The recruitment of inflammatory cells into the gut wall probably is secondary to the damage caused by the ethanol.

At 1 h after administration of a single dose of TNBS in ethanol in the colon descendens, oedema and hyperaemia is already present in the gut wall, as well as small ulcerative areas. Macroscopical damage scores (Table 1) increase to a maximum on $t = 24$ h, and gradually improve after $t = 72$ h. In the mucosa the percentage of macrophages gradually increases from 1 h till day 7, whereas mucosal neutrophils showed only a slight increase. However, in the submucosa a striking increase of macrophages is found at 24 h and neutrophils show the highest numbers at 72 h (personal observation). During this very acute stage of the inflammatory reaction, no significant differences were found between rats that were treated with ethanol alone or TNBS in ethanol. These data illustrate that in this model macrophages are the first leukocytes that migrate into the already damaged gut wall, prior to the increase of neutrophils, and are probably responsible for the increased macroscopical damage score at

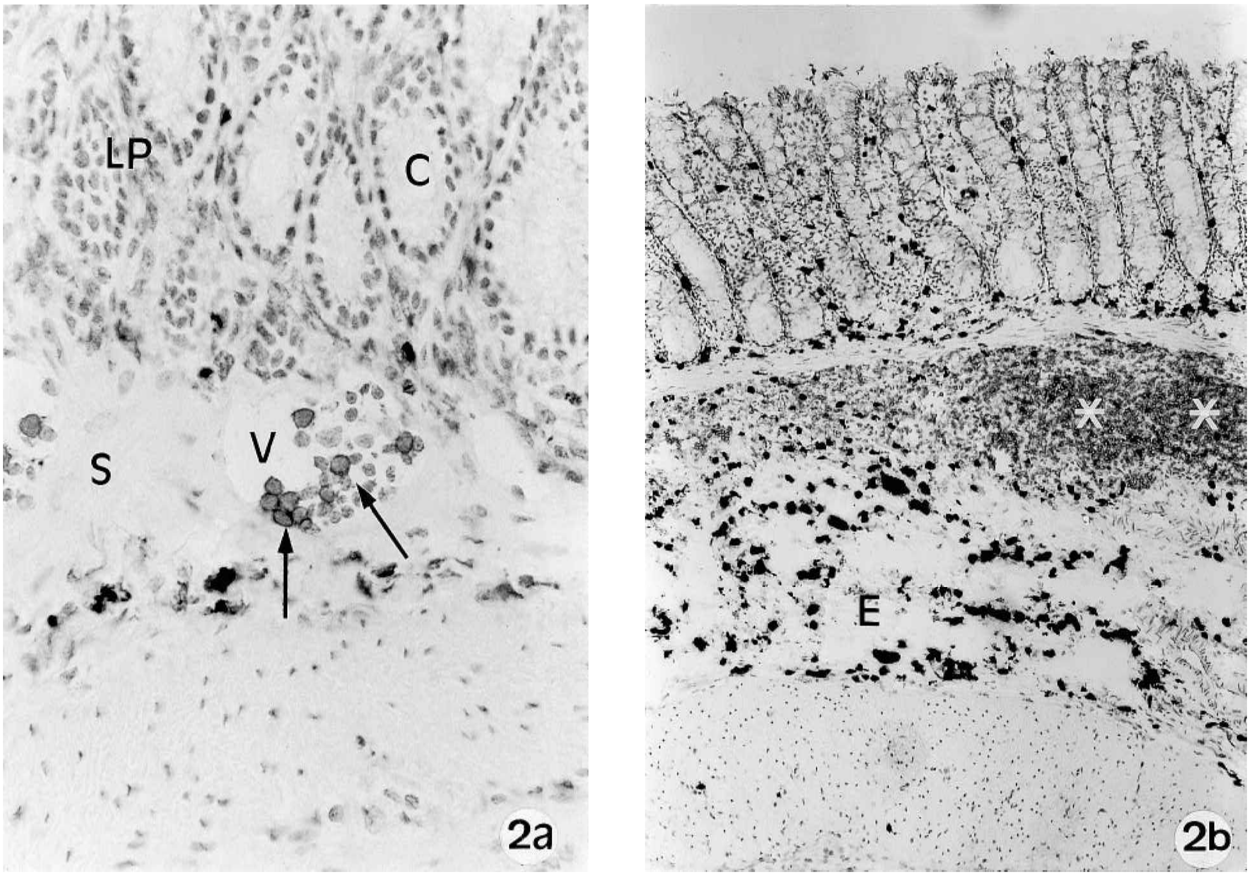

FIG. 2. T lymphocytes in TNBS/ethanol-induced colitis. (A) In the chronic phase of the disease T cells play a more important role than in the acute phase. This section shows T cells in a submucosal blood vessel (arrows). Staining with mAb OX19, recognizing rat T cells. LP = lamina propria; C = crypts; V = vessel; S = submucosa. (B) Three days after a transfer of splenic CD4+ cells, obtained from a rat that suffered from TNBS/ethanol-induced colitis, into a naive recipient. Large T cell infiltrates are present (asterisks). Note the presence of oedema and the increase of small, black neutrophils (arrows).

**Table 1**. Macroscopical damage score

| | |
|---|---|
| 0 | no damage |
| 1 | localized hyperaemia and/or oedema |
| 2 | linear ulcer < half of the width of the colon |
| 3 | linear ulcer > half of the width of the colon or small circular ulcer with a diameter < 1 cm |
| 4 | 2 circular ulcers with a diameter < 1 cm |
| 5 | circular ulcer with a diameter between 1 and 2 cm |
| 6 | circular ulcer with a diameter > 2 cm |
| 7 | circular ulcerative area with a diameter > 4 cm |

$t = 24$ h. In another study on early leukocyte influx in this model no data were obtained on increase of macrophages.[4] After day 14, no significant differences are present anymore between rats that had received only ethanol in the colon, and healthy control rats. This indicates that ethanol in itself induces an acute inflammation. In TNBS/ethanol-induced inflammation a more chronic stage follows after day 14, which lasts about 8–12 weeks.

Macrophages and dendritic cells play an important role in active IBD. Large numbers of macrophages can be detected in the colon of patients with CD.[5,6] Furthermore, differences are found, not only in number but also in heterogeneity of macrophage subpopulations and dendritic cells in colon from IBD patients compared with healthy controls and compared with non-IBD intestinal inflammation.[5,7] After treatment of the disease the appearances returned to normal, indicating that the changes seen in macrophage subpopulations in IBD are not primary to the disease itself.[8] Tissue concentrations of macrophage derived pro-inflammatory cytokines correlate with disease activity.[9]

In rat colon, lymphocytes are normally present in small numbers, but after TNBS/ethanol induced colitis a slight increase was found in the number of B cells and a striking increase was observed in the number of T cells in the submucosa, especially between day 14 and day

28. The importance of T cells in experimental colitis has been described before by Sartor, who observed that the chronic stage of peptidoglycan–polysaccharide complex (PG-PS)-induced enterocolitis is T-cell dependent.[10]

In an attempt to induce a relapse in the TNBS/ethanol model, a second, extra-intestinal administration of TNBS (without ethanol) was given after the initial exposure of the colon to TNBS/ethanol.[3] This resulted in new areas of ulceration and renewed increase of leukocytes in the colonic tissue and these data suggest that the rats had been sensitized by the first dose of TNBS in the colon.

### Selectins in Inflammatory Bowel Disease

Selectins are cell adhesion molecules whose carbohydrate binding domain is involved in leukocyte adhesion to activated vascular endothelium. They play a pivotal role in the early steps of the inflammatory process mediating the attachment of flowing leukocytes to the endothelial cells with labile adhesions in the direction of the blood flow.[11] The initial interaction is represented by physical rolling of the leukocyte along the vessel wall. The loose connection and disconnection seems to be mediated through rapid association and dissociation rate constants.[12] The tethering interaction allows the leukocyte to respond to subsequent signalling events that induce tight adhesion which is necessary for transendothelial migration. Once neutrophils tightly adhere to endothelial cells, the bound neutrophil mediates the rolling of newly arriving neutrophils.[13] Recently it has been shown that T lymphocytes as well, after being bound to endothelial cells, support the rolling of newly arrived T lymphocytes.[14] The selectin-mediated step, prior to firm adhesion to the endothelial cells is a prerequisite for the subsequent chemoattractant- and integrin-mediated steps.

Selectins are a family of three proteins. P-selectin is stored in granules of platelets and endothelial cells and is expressed on the surface of these cells within minutes of cellular stimulation. The expression of E-selectin on the surface of endothelial cells occurs over a period of hours following cellular activation. L-selectin is constitutively expressed on most lymphocytes and mediates trafficking from the blood to lymphatics via high endothelial venules. Selectins have a multidomain structure which includes a signal sequence, transmembrane domain, epidermal growth factor (EGF)-like domain, a series of consensus repeats related to complement regulatory domains and a carbohydrate-binding (lectin) domain. It is this latter domain that is responsible for the adhesion function via binding to a carbohydrate ligand on leukocytes.

An increased expression of P-selectin is present on veins, venules and capillaries in highly inflamed gut, without differences between CD and UC.[15] In uninvolved gut, P-selectin expression was similar to that seen in normal controls, except for a focal increase of P-selectin in the vicinity of small lymphocyte aggregates.[15] E-selectin is present in tissue in active CD and UC as well, sulphasalazine decreased E-selectin expression.[16] As within CD and UC no difference in soluble E-selectin in the serum was found between the active and inactive states,[17] serum levels of selectins do not seem to be useful as a marker for disease activity.

Small synthetic peptides, based on a conserved region of E-, L- and P-selectins inhibited neutrophil adhesion and infiltration *in vitro* and *in vivo*.[18,19] Selectin-derived peptides were shown to inhibit leukocyte trafficking into thioglycollate-treated peritoneum.[19] Inhibition of neutrophil infiltration into IL-1$\alpha$ treated skin also has been found following treatment with selectin derived peptides.[19] This effect is probably mediated by blocking the carbohydrate ligand for selectins of leukocytes.

Based on this study, a seven amino acid peptide with the sequence YYWIGIR-$NH_2$ (Tyr-Tyr-Trp-Ile-Gly-Ile-Arg-$NH_2$) has been studied in TNBS/ethanol induced colitis (personal observations).[20] Treatment with YYWIGIR-$NH_2$, based on the sequence of the lectin domain of selectins, has a beneficial effect in TNBS/ethanol induced colitis in rats provided treatment takes place during a period of 7 days. The treatment resulted in a significant improvement of macroscopical damage scores (Table 1) with 70%, compared with the saline-treated colitis rats (Fig. 3). Regarding the histopathology, most animals had full re-epithelialization after 7 days of treatment. Administration of the same peptide during 3 days did have a slight improving effect on macroscopical damage scores but not on histopathological, microscopical scores. This means that the actual area of ulceration was slightly smaller after 3 days of treatment but that the inflamed areas in themselves were as severely affected as saline-treated rats.

After treatment with YYWIGIR-$NH_2$ for 7 days, the number of neutrophils in the submucosa was significantly reduced. No influence was found on macrophages either in the mucosa or the submucosa of the peptide treated animals. MPO activity was decreased significantly in the

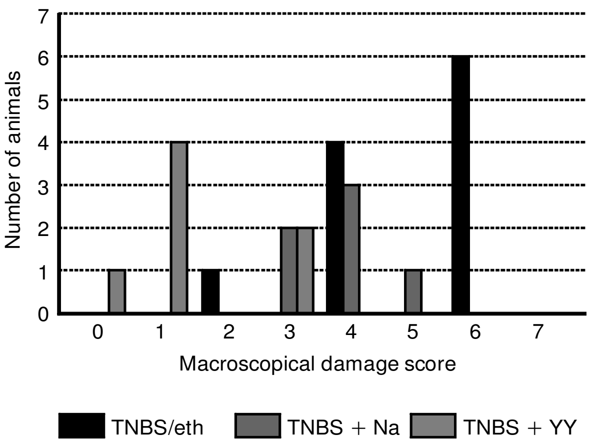

FIG. 3. Treatment of experimental colitis in rats with selectin-derived peptides: influence on the distribution pattern of macroscopical damage scores on day 7 after induction. TNBS/eth = rats that only received an enema with TNBS/ethanol, no further treatment. TNBS + Na: rats that were treated with vehicle, after induction of colitis. TNBS + YY: rats were treated with the selectin-derived peptide YYWIGIR-$NH_2$ on days 0–7 after induction of colitis.

colon of the rats that had been treated with YYWIGIR-$NH_2$ on day 0–7 after induction of colitis, in accordance with the decreased numbers of neutrophils in the submucosa.

In earlier studies describing the effects of this peptide on various inflammatory conditions, it was suggested that its anti-inflammatory effect may be mediated by blocking the carbohydrate ligand for the (endothelial) selectins on the surface of the leukocytes.[19] In line with this hypothesis, YYWIGIR-$NH_2$ may be capable of blocking the infiltration of circulating neutrophils into colonic submucosa. However, monocytes also have carbohydrate ligands to E- and P-selectin, and yet the number of macrophages in the inflamed colon was not decreased by YYWIGIR-$NH_2$. In the previous papers studying selectin-derived peptides, emphasis was put on the migration of neutrophils and hence no data are available yet on the effect of the peptides on the trafficking of other leukocytes, such as monocytes and lymphocytes. The differential in effect on the increase of neutrophils and macrophages may be due to a difference in affinity of the carbohydrate selectin-ligand to YYWIGIR-$NH_2$. It cannot be excluded that higher doses would result in a decrease of macrophages as well.

Recently it has been described that whereas many single amino acid substitutions are tolerated in the peptide structure without complete loss of inhibitory activity, substitutions at some positions (e.g. the W residue) results in relatively inactive compounds.[21] This points at the importance of these residues in making critical contacts with the appropriate saccharide ligand.[21]

### Chemokines in Inflammatory Bowel Disease

Chemoattractants play a pivotal role in the activation of integrin adhesiveness and in directing the migration of leukocytes. Leukocytes move in the direction of the chemoattractant. A family of chemoattractive cytokines, which is termed the chemokine family,[22] are 70–80 residue polypeptides and have specificity for leukocyte subsets.[23,24] Their ability to attract and activate leukocytes makes them important inflammatory mediators. All chemokines have four conserved cysteines, and two subfamilies have been identified based on a difference in sequence around two cysteine residues. The members of these two families differ in their target cell selectivity. The C-X-C or $\alpha$ chemokines in particular attract neutrophils whereas the C-C or $\beta$ chemokines primarily act on monocytes and T lymphocyte subpopulations. The specificity of the chemokine subfamilies seems to be regulated by the expression of their receptors on the target cells.[2] An important member of the C-X-C subfamily is IL-8; members of the C-C chemokine subfamily are monocyte-chemoattractant protein 1 (MCP-1) and RANTES.

Regarding chemokines in IBD, several studies have focused on IL-8 in intestinal inflammation. Affected colonic mucosa obtained from patients with active CD or UC contains significantly more IL-8 than tissue of controls,[25] but also more IL-8 than patients with inactive disease.[26] The production of IL-8 mRNA is restricted to areas with histological signs of intestinal inflammation in IBD but also in non-IBD inflammation.[27] In IBD, neutrophils and recently recruited CD14+ macrophages are responsible for the production of IL-8.[28] Epithelial cells in normal and inflamed tissue showed neither mRNA for IL-8 nor protein. IL-8 mRNA was expressed significantly more commonly by macrophages from IBD affected than from normal mucosa, and IL-8 secretion by IBD but not normal colon macrophages was augmented by lipopolysaccharide treatment. These data suggest a continuous cycle of neutrophil activation in IBD[28] with an important role for bacterial antigens derived from the gut lumen.

Serum IL-8 is enhanced in some IBD patients groups, but in CD no difference was found between active and inactive disease.[29] These data suggest that although IL-8 as a local chemoattractant is involved in the inflammatory process in the intestine, it is a poor marker of disease activity.[29]

Regarding the C-C chemokines, MCP-1 is expressed constitutively in the human intestinal colonic mucosa and is upregulated during inflammation. In addition to lamina propria macrophages, endothelial cells and intestinal epithelial cells are able to produce MCP-1.[30] In another study, MCP-1 gene expression was restricted to the lamina propria in IBD-patients, whereas the gene encoding for RANTES was expressed in intra-epithelial lymphocytes and in the sub-epithelial lamina propria.[31] Compared with non-inflamed controls, in endothelial cells of venules in IBD an increase of MCP-1 mRNA and an increase of MCP-1 expressing cells was observed.[31,32] As no increase in mRNA was found for RANTES in endothelial cells,[31] this suggests a pivotal role for MCP-1 but not for RANTES in the adhesion of blood monocytes in IBD.[31] RANTES is being produced by T lymphocytes and besides monocytes has other T lymphocytes as its target cells.[2] Taken together with the described epithelia-associated localization, this suggests a role for RANTES in directing local T lymphocytes and monocytes towards the epithelial layer which is disrupted and vulnerable to lumenal bacteria in IBD.

In experimentally induced colitis, the first leukocytes that increase significantly after intracolonic administration of TNBS in ethanol are macrophages, whereas neutrophils do not increase in number until $t = 48$ h after the induction of intestinal inflammation (personal observation). This early involvement of macrophages may suggest that upregulation of MCP-1 precedes the production of neutrophil attracting C-X-C chemokines such as IL-8.

### Integrins in Inflammatory Bowel Disease

A family of adhesion glycoproteins which mediate firm leukocyte adherence to the vascular endothelium is the family of the integrins. Each integrin has a non-covalently associated α and β subunit. Several integrins are important in the interaction of leukocytes with endothelial cells such as the β2-integrins or CD11/CD18. CD11/CD18 is a heterodimeric complex consisting of non-covalently associated α and β units. Three forms of the α component of the adhesion molecule have been identified: αL (CD11a or lymphocyte function-associated antigen LFA-1), αM (CD11b or complement receptor type 3, CR3) and αX (CD11c). Each α component is associated with a common β2 subunit CD18. Two other members of the integrin family share the same α subunit: the α4-integrins α4β1 (VLA-4) and α4β7 (LPAM-1).

The β2-integrins are all expressed on monocytes and neutrophils, although α1β2 is also expressed on lymphocytes. The ligands for these leukocyte integrins are intercellular adhesion molecules (ICAM)-1,2 and 3. Induction of ICAM-1 on endothelial cells by pro-inflammatory cytokines may increase leukocyte adhesion and migration into inflammatory sites, whereas the constitutive expression of ICAM-2 may be important in leukocyte trafficking in uninflamed sites.[2] The α4-integrins are mainly expressed on B and T lymphocytes, and they all play a role in adhesion and migration of leukocytes to the endothelium.[2] Vascular cell adhesion molecule 1 (VCAM-1) is a ligand for α4β1. α4β7 is the receptor for MadCAM-1 which is an adhesion molecule for lymphocytes that is expressed by mucosal venules and helps direct lymphatic traffic into Peyer's patches and the intestinal lamina propria. The interaction of α4β7 and MadCAM-1 plays a role in lymphocyte homing to mucosal sites.[33]

Patients with active CD or UC have significantly higher expression of ICAM-1 on venules[34] and higher ICAM-1 in serum as well.[35,36] In several studies it has been shown that mAbs against adhesion molecules reduce migration of leukocytes into inflammatory sites. Treatment with mAbs against ICAM-1, started after the onset of colitis in rats, had a beneficial effect.[37] mAbs directed against the common β2 component of the integrins are capable of preventing adherence through all three members of the integrin family. In animal models, anti-CD18 has been shown to reduce perfusion injury in various tissues and species.[38–40] Furthermore, anti-CD18 reduced indomethacine-induced gastropathy[41] and TNBS/ethanol induced experimental colitis in rabbits.[42] Treatment of TNBS/ethanol-induced colitis with anti-CD11b/CD18 mAbs results in reduced influx of neutrophils and macrophages into the colon, which was shown by histology and by reduced MPO activity.[43] In the latter study, levels of MPO activity were lower than was to be expected based upon the reduced numbers of inflammatory cells: this reduction was 50–85%, whereas the MPO activity was almost absent. This might be explained by the fact that anti-CD11b/CD18 mAbs not only reduce migration of inflammatory cells but also influence phagocyte functions that are thought to play a role in inflammation. CD11/CD18 integrins are required for neutrophils to initiate a respiratory burst in response to soluble cytokines.

### T Lymphocyte Migration in Intestinal Inflammation

In rodents and rabbits, administration of TNBS in ethanol results in an acute colonic inflammation.[1] From 2–4 weeks after the induction of colitis an increase in the number of T cells is observed in the colon (Fig. 2A). The importance of T cells in this model was shown by Selve and Wohrmann[44] who described the sensitization of rats by an intradermal injection of TNBS, prior to application of TNBS/ethanol to the colon, resulting in a more severe inflammation when TNBS/ethanol was given. Furthermore, it is also possible to induce tolerance prior to the application of TNBS/ethanol in the colon, resulting in a reduced inflammation.[45] These results show that the chronic inflammation is not based on a non-specific immune response, in contrast to the acute phase.

It has been suggested that TNBS/ethanol-induced colitis in mice is mediated by a Th1 response, since the production of IFNγ and IL-2 were upregulated in lamina propria $CD4^+$ cells obtained from these mice.[46] This Th1 cytokine pattern is also found in other animal models.[9] Evidence is accumulating in favour of a Th1-like response in Crohn's disease.[47]

Relapses are an important feature of human disease in IBD. In the TNBS/ethanol model spontaneous relapses do not occur, but it is possible to induce relapses by a single or multiple extra-intestinal boosters of TNBS.[3,48] To further elucidate the role of T cells in this model, splenic T cells from colitis rats were transferred to naive recipients.[49] Transfer of $CD4^+$ T cells to naive recipients results in migration of the transferred cells to all lymphoid organs, but in particular to the colonic submucosa on day 1–2 after the transfer. These data suggest that the T cells that migrate to the colon are memory T cells that had been sensitized before in the donor colon following the TNBS/ethanol application. At subsequent days also recipients' own T cells migrate to the colon, and neutrophils and macrophages also increase in the colonic submucosa (Fig. 2B). These cells probably are directed to the colon by cytokine/chemokine signals that are either produced or induced by the T cells. There are several ways to explain the migration of memory T cells to the colon. Firstly, there might be a difference in expression of adhesion molecules and chemokines, such as RANTES that in particular attracts memory T lymphocytes,[50] between colon and other tissues including the small bowel, although there is no proof of such a difference within the intestinal tract yet. Secondly, the transferred T cells might recognize a colon-specific hapten-modified self-antigen. Another explanation is that the transferred T cells recognize a component of the recipients bacterial flora in the intestine. It has been suggested that, even in healthy immunocompetent hosts, indigenous bacteria are continuously translocating in low numbers from the luminal side to the gut tissue, mesenteric lymph nodes and other extra-intestinal organs.[51] Under physiological conditions the translocated bacteria are engulfed and killed by macrophages in the colonic tissue and in the lymph nodes. In case of enhanced bacterial translocation and an impaired immune reactivity this will lead to further passage from mesenteric lymph nodes to liver, spleen, kidney, peritoneal cavity and the blood. Eventually this may lead to sepsis.[52] Upon induction of colitis, T cells may be sensitized by bacterial components. Transfer of these T cells to recipients may result in recognition of similar bacterial components[43] since there is always some translocation into the intestinal wall.[51]

Migration of lymphocytes into the gut is also mediated by sequential groups of adhesion molecules, with selectin- and integrin-mediated steps. Lymphocytes that interact with vascular selectins represent distinct subsets. Certain memory T cells bind and roll on E-selectin.[53,54] Both E- and P-selectin support rolling interactions of bovine γδ T cells under physiologic flow.[55] γδ lymphocytes, bound to an endothelial cell monolayer, support rolling of other lymphocytes. Anti-L-selectin mAbs block this interaction.[14]

However, lymphocyte homing to the inflamed gut seems to be regulated differently from lymphocyte homing into non-inflamed gut tissue. Selective migration of lymphocytes is mediated by organ-specific binding of lymphocytes to high endothelial venules (HEV). Functionally distinct lymphocyte–endothelial recognition systems exist in the mucosa-associated lymphatic tissues, peripheral lymph node, synovium, and skin.[56–58] A difference in β7 (Peyer's patch) specific antigen was observed between lymphocytes obtained from IBD patients and healthy controls.[59] Moreover, it has been shown that lamina propria immunoblasts from normal and inflamed gut bind differently to vascular endothelium.[60] Immunoblasts from the normal gut bound extremely well to mucosal HEV but not to peripheral lymph node HEV, whereas cells obtained from IBD patients also interacted with peripheral lymph node HEV. In IBD patients the peripheral lymph node-specific endothelial adhesion antigen was aberrantly expressed in vessels of the inflamed mucosa.[60] Therefore, in IBD the selectivity of lymphocyte–endothelial interaction seems to be lost.

### References

1. Morris GP, Beck PL, Herridge MS, Depew WT, Szewczuk MR, Wallace JL. Hapten-induced model of chronic inflammation and ulceration in the rat colon. *Gastroenterology* 1989; **96**: 795–803.
2. Springer TA. Traffic signals for lymphocyte recirculation and leukocyte emigration: the multistep paradigm. *Cell* 1994; **76**: 301–314.
3. Palmen MJHJ, Dieleman LA, van der Ende MB, Uyterlinde A, Peña AS, Meuwissen SGM, van Rees EP. Non-lymphoid and lymphoid cells in acute, chronic and relapsing experimental colitis. *Clin Exp Immunol* 1995; **99**: 226–232.
4. Yamada T, Marshall S, Specian RD, Grisham M. A comparative analysis of two models of colitis in rats. *Gastroenterology* 1992; **102**: 1524–1534.
5. Wilders MM, Drexhage HA, Kokje M, Verspaget HW, Meuwissen SGM. Veiled cells in chronic idiopathic inflammatory bowel disease. *Clin Exp Immunol* 1984; **55**: 461–468.
6. Seldenrijk CA, Drexhage HA, Meuwissen SGM, Pals ST, Meijer CJ. Dendritic cells and scavenger macrophages in inflammatory bowel disease. *Gut* 1989; **30**: 484–491.
7. Alison MC, Poulter LW. Changes in phenotypically distinct mucosal macrophage populations may be a prerequisite for the development of inflammatory bowel disease. *Clin Exp Immunol* 1991; **85**: 504–509.
8. Selby WS, Poulter LW, Hobbs S, Jewell DP, Janossy G. Heterogeneity of HLA-DR-positive histiocytes in human intestinal lamina propria: a combined histochemical and immunohistological analysis. *J Clin Path* 1983; **36**: 379–384.
9. Sartor RB. Cytokines in intestinal inflammation: pathophysiological and clinical considerations. *Gastroenterology* 1994; **106**: 533–539.
10. Sartor RB, Bender DE, Allen JB, *et al.* Chronic experimental entercolitis and extra-intestinal inflammation are T lymphocyte dependent. *Gastroenterology* 1993; **104**: A775.
11. Lawrence MB, Springer TA. Leukocytes roll on a selectin at physiologic flow rates: distinct from and prerequisite for adhesion through integrins. *Cell* 1991; **65**: 859–873.
12. Ushiyama S, Laue TM, Moore KL, Erickson HP, McEver RP. Structural and functional characterization of monomeric soluble P-selectin and comparison with membrane P-selectin. *J Biol Chem* 1993; **268**: 15229–15237.
13. Bargatze RF, Kurk S, Butcher EC, Jutila MA. Neutrophils roll on adherent neutrophils bound to cytokine-induced endothelial cells via L-selectin on the rolling cells. *J Exp Med* 1994; **180**: 1785–1790.
14. Jutila MA, Kurk S. Analysis of bovine γδ T cell interactions with E-, P-, and L-selectin. *J Immunol* 1996; **156**: 289–296.
15. Schurmann GM, Bishop AE, Facer P, Vecchio M, Lee JC, Rampton DS, *et al.* Increased expression of cell adhesion molecule P-selectin in active inflammatory bowel disease. *Gut* 1995; **36**: 411–418.
16. Pooley N, Ghosh L, Sharon P. Up-regulation of E-selectin and intercellular adhesion molecule-1 differs between Crohn's disease and ulcerative colitis. *Dig Dis Sci* 1995; **40**: 219–225.
17. Jones SC, Banks RE, Gearing AJ, Hemingway IK, Ibbotson SH, Dixon MF, Axon AT. Adhesion molecules in inflammatory bowel disease. *Gut* 1995; **36**: 724–730.
18. Geng JG, Heavner GA, McEver RP. Lectin domain peptides from selectins interact with both cell surface ligands and $Ca^{2+}$ ions. *J Biol Chem* 1992; **267**: 19846–19853.
19. Briggs JB, Oda Y, Gilbert JH, Schaefer ME, Macher BA. Peptides inhibit selectin-mediated cell adhesion in vitro, and neutrophil influx into inflammatory sites in vivo. *Glycobiology* 1995; **5**: 583–588.
20. Van Rees EP, Van der Goot FRW, Palmen MJHJ, Macher BA. Peptide inhibitor of selectin-mediated cell adhesion has a beneficial effect in experimental IBD. *Gastroenterology* 1996; **110**: A1036.
21. Briggs JB, Larsen RA, Harris RB, Sekar KVS, Macher BA. Structure/activity studies of anti-inflammatory peptides based on a conserved peptide region of the lectin domain of E-, L- and P-selectin. *Glycobiology* 1996; **6**: 831–836.
22. Lindley IJD, Westwick J, Kunkel SL. Nomenclature announcement: the chemokines. *Immunol Today* 1993; **14**: 24.
23. Oppenheim JJ, Zachariae COC, Mukaida N, Matsushima K. Properties of the novel proinflammatory supergene 'intercine' cytokine family. *Annu Rev Immunol* 1991; **9**: 617–648.
24. Miller MD, Krangel MS. Biology and biochemistry of the chemokines: a family of chemotactic and inflammatory cytokines. *Crit Rev Immunol* 1992; **12**: 17–46.
25. Izzo RS, Witkon K, Chen AI, Hadjiyane C, Weinstein MI, Pellecchia C. Interleukin-8 and neutrophil markers in colonic mucosa from patients with ulcerative colitis. *Am J Gastroent* 1992; **87**: 1447–1452.
26. Mitsuyama K, Toyonaga A, Sasaki E, *et al.* IL-8 as an important chemoattractant for neutrophils in ulcerative colitis and Crohn's disease. *Clin Exp Immunol* 1994; **96**: 432–436.
27. Mazzucchelli L, Hauser C, Zgraggen K, Wagner H, Hess M, Laissue JA, Mueller C. Expression of interleukin-8 gene in inflammatory bowel disease is related to the histological grade of active inflammation. *Am J Path* 1994; **144**: 997–1007.
28. Grimm MC, Elsbury SK, Pavli P, Doe WF. Interleukin 1: cells of origin in inflammatory bowel disease. *Gut* 1996; **38**: 90–98.
29. Jones SC, Evans SW, Lobo AJ, Ceska M, Axon AT, Whicher JT. Sum interleukin-8 in inflammatory bowel disease. *J Gastroenterol Hepatol* 1993; **8**: 508–512.
30. Reinecker HC, Loh EY, Ringler DJ, Mehta A, Rombeau JL, McDermott RP. Monocyte-chemoattractant protein 1 gene expression in intestinal epithelial cells and inflammatory bowel disease mucosa. *Gastroenterology* 1995; **108**: 40–50.
31. Mazzucchelli L, Hauser C, Zgraggen K, Wagner HE, Hess MW, Laissue JA, Mueller C. Differential in situ expression of the genes encoding the chemokines MCP-1 and RANTES in human inflammatory bowel disease. *J Pathology* 1996; **178**: 201–206.
32. Grimm MC, Elsbury SK, Pavli P, Doe WF. Enhanced expression and production of monocyte chemoattractant protein-1 in inflammatory bowel disease mucosa. *J Leukocyte Biol* 1996; **59**: 804–812.
33. Berlin C, Berg EL, Briskin MJ, *et al.* α4β7 integrin mediates lymphocyte binding to the mucosal vascular addressin MAdCAM-1-1. *Cell* 1993; **74**: 185–195.
34. Nakamura S, Ohtani H, Watanabe Y, *et al.* In situ expression of the cell adhesion molecules in inflammatory bowel disease: evidence of immunologic activation of vascular endothelial cells. *Lab Invest* 1993; **89**: 77–85.
35. Jones SC, Banks RE, Haider A, *et al.* Adhesion molecules in inflammatory bowel disease. *Gut* 1995; **36**: 724–730.
36. Patel RT, Pall AA, Adu D, Keighley MR. Circulating soluble adhesion molecules in inflammatory bowel disease. *Eur J Gastroenterol Hepatol* 1995; **7**: 1037–1041.
37. Wong PY, Yue G, Yin K, Miyasaka M, Lane CL, Manning AM, Anderson DC, Sun FF. Antibodies to intercellular adhesion molecule-1 ameliorate the inflammatory response in acetic acid-induced inflammatory bowel disease. *J Pharmacol Exp Ther* 1995; **274**: 475–480.
38. Horgan MJ, Wright DS, Malik AB. Antibody against leukocyte integrin (CD 18) prevents reperfusion-induced lung vascular injury. *Am J Physiol* 1990; **259**: L315–L319.
39. Vedder NB, Winn RK, Rice CL, Chi EY, Arfors KE, Harlan JM. Inhibition of leukocyte adherence by anti-CD18 monoclonal antibody attenuates reperfusion injury in the rabbit ear. *Proc Natl Acad Sci* 1990; **87**: 2643–2646.
40. Mileski WJ, Winn RK, Vedder NB, Pohlman TH, Harlan JM, Rice CL. Inhibition of CD18-dependent neutrophil adherence reduces organ injury after hemorrhagic shock in primates. *Surgery* 1990; **108**: 206–212.
41. Wallace JL, Arfors KE, McKnight GW. A monoclonal antibody against the CD 18 leukocyte adhesion molecule prevents indomethacin-induced gastric damage in the rabbit. *Gastroenterology* 1991; **100**: 878–883.
42. Wallace JL, Higa A, McKnight GW, MacIntyre DE. Prevention and reversal of experimental colitis by a monoclonal antibody which inhibits leukocyte adherence. *Inflammation* 1992; **16**: 343–354.
43. Palmen MJHJ, Dijkstra CD, van der Ende MB, Peña AS, van Rees EP. Anti-CD11b/CD18 antibodies reduce inflammation in acute colitis in rats. *Clin Exp Immunol* 1995; **101**: 351–356.
44. Selve N, Wohrmann T. Intestinal Inflammation in TNBS sensitized rats as a model of chronic inflammatory bowel disease. *Med Inflamm* 1992; **1**: 121–126.
45. Beck PL, Moris GP, Wade AW, Szewczuk M, Wallace JL. Immunological manipulation of disease progression in a rat model of chronic inflammatory bowel disease of the colon. In: McDermott R, ed. *Inflammatory Bowel Disease: Current Status and Future Approach*. Amsterdam: Elsevier Science Publishers, 1988: 201–206.
46. Neurath MF, Fuss I, Kelsall BL, Stuber E, Strober W. Antibodies to interleukin 12 abrogate established experimental colitis in mice. *J Exp Med* 1995; **182**: 1281–1290.
47. Mullin GE, Vezza FR, Sampat A, *et al.* Abnormal IL-10 mRNA production in the intestinal mucosal lesions in inflammatory bowel disease. *Gastroenterology* 1993; **104**: A751.
48. Appleyard C, Wallace JL. Reactivation of hapten-induced colitis and its prevention by anti-inflammatory drugs. *Am J Physiol* 1995; **269**: 119–125.
49. Palmen MJHJ, Wijburg OLC, Kroes H, Peña AS, Van Rees EP. Hapten-induced experimental colitis is transferable by extra-intestinal CD4+ T cells. (Submitted).
50. Schall TJ, Bacon K, Toy KJ, Goeddel DV. Selective attraction of monocytes and T lymphocytes of the memory phenotype by cytokine RANTES. *Nature* 1990; **347**: 669–671.
51. Berg RD. Bacterial translocation from the gastrointestinal tract. *Trends Microbiol* 1995; **3**: 149–154.
52. Berg RD, Wommack E, Deitch EA. Immunosuppression and intestinal bacterial overgrowth synergistically promote bacterial translocation. *Arch Surg* 1988; **123**: 1359–1364.

53. Alon R, Rossiter H, Wang X, Springer TA, Kupper TS. Distinct cell surface ligands mediate T lymphocyte attachment and rolling on P- and E-selectin under physiological low. *J Cell Biol* 1994; **127**: 1485–1495.
54. Jones DA, McIntire LV, Smith CW, Picker LJ. A two-step adhesion cascade for T cell/endothelial cell interactions under flow conditions. *J Clin Invest* 1994; **94**: 2443–2450.
55. Jutila MA, Bargatze RF, Kurk S, Warnock RA, Ehsani N, Watson SR, Walchek B. Cell surface P- and E-selectin support shear-dependent rolling of bovine γδ T cells. *J Immunol* 1994; **153**: 3917–3928.
56. Butcher EC, Scollay RG, Weissman IL. Organ specificity of lymphocyte homing: mediation by highly selectively lymphocyte interaction with organ-specific determinants on high endothelial venules. *Eur J Immunol* 1980; **10**: 556–561.
57. Jalkanen S, Steere AC, Fox RI, Butcher EC. A distinct endothelial recognition system that controls lymphocyte traffic into inflamed synovium. *Science* 1986; **233**: 556–560.
58. Picker LJ, Kishimoto TK, Smith CW, Warnock RA, Butcher EC. ELAM-1 is an adhesion molecule for skin-homing T cells. *Nature* 1991; **349**: 796–799.
59. Yacyshyn BR, Lazarovits A, Tsai V, Matejko K. Crohn's disease, ulcerative colitis, and normal intestinal lymphocytes express integrins in dissimilar pattern. *Gastroenterology* 1994; **107**: 1364–1371.
60. Salmi M, Granfors K, MacDermott R, Jalkanen S. Aberrant binding of lamina propria lymphocytes to vascular endothelium in inflammatory bowel diseases. *Gastroenterology* 1994; **106**: 596–605.

**Received 18 October 1996;**
**accepted in revised form 16 December 1996**
